# Update on policy change proposal to end Institutional Biosafety Committee (IBC) review of rDNA vaccine clinical trials

**DOI:** 10.1186/1742-4690-9-S2-P241

**Published:** 2012-09-13

**Authors:** ME Enama, N Jones, BS Graham, HC Lane

**Affiliations:** 1 VRC/NIAID/NIH, Bethesda, MD, USA; 2 OSPFM/NIAID/NIH, Bethesda, MD, USA; 3 DCR/NIAID/NIH, Bethesda, MD, USA

## Background

The HIV vaccine regimen showing partial efficacy in the RV 144 study included a canarypox-vectored vaccine (ALVAC). The ongoing HIV vaccine efficacy trial, HVTN 505, involves two gene-based vaccines developed by VRC/NIAID/NIH: plasmid DNA and adenoviral-vectored vaccines. The “NIH Guidelines for Research Involving Recombinant DNA Molecules,” established in 1976, have evolved over time. Current NIH policy is that every clinical trial of rDNA vaccines must have IBC reviews. The primary role of the IBC is to assess risk to public health and the environment.

## Methods

The NIAID Barriers to Clinical Research project identified repetitive IBC review of gene-based vaccines as a barrier. For example, in the last 10 years >100 local IBC reviews have been conducted for the VRC HIV vaccines. IBC reviews for ALVAC vaccines remain a requirement for NIH funding despite completion of a Phase III clinical trial, >20 years of human experience with ALVAC vaccines and widespread community-based use of USDA-approved canarypox-vectored veterinary vaccines, which have not indicated a risk to public health or the environment.

## Results

The NIAID policy change proposal has been considered by a Recombinant DNA Advisory Committee (RAC) working group. Policy change options were discussed at the September 2011, December 2011 and March 2012 RAC meetings. The RAC proposal will be published in the Federal Register for public comments.

## Conclusion

Repetitive IBC reviews of rDNA vaccine clinical trials divert time and attention of IBC expertise from truly novel agents and incur a cost to NIH with no added safety benefit. NIAID proposes that IBC review is no longer needed for clinical trials of non-transmissible rDNA vaccines because there are no unique biosafety concerns for this class of vaccines based on their recombinant DNA nature. The IBC specific roles are not applicable for rDNA vaccine protocols and are fulfilled through other regulatory oversight including the FDA.

